# Applying a logistic regression-clustering joint model to analyze the causes of prolonged pre-analytic turnaround time for urine culture testing in hospital wards

**DOI:** 10.3389/fdgth.2025.1603314

**Published:** 2025-06-30

**Authors:** Shuangshuang Lv, Huan Ye, Yuan Li, Jian Zhang

**Affiliations:** ^1^ Clinical Laboratory, Dongyang People’s Hospital, Dongyang, Zhejiang, China; ^2^ Clinical Laboratory, The Second People’s Hospital of Yuhuan City, Yuhuan, Zhejiang, China

**Keywords:** logistic regression model, urine microbial culture, pre-analytical turnaround time (pre-TAT), medical quality control, process optimization

## Abstract

**Introduction:**

In this study, we developed and validated a logistic regression-clustering joint model to: (1) quantify multistage workflow bottlenecks (collection/transport/reception) in urine culture pre-TAT prolongation (>115 min); and (2) assess the efficacy of targeted interventions derived from model-derived insights.

**Methods:**

Using complete workflow data obtained from 1,343 urine culture specimens (January 2024–March 2024) collected at a tertiary hospital, we integrated binary logistic regression analysis with K-means clustering to quantify delay patterns. The analyzed variables included collection time, ward type, personnel roles, and patient demographics. Post-intervention data (May 2024–July 2024, *n* = 1,456) was also analyzed to assess the impact.

**Results:**

Analysis of the critical risk factors revealed that specimens collected between 04:00–05:59/10:00–11:59 had 142.92-fold higher delay odds (95% CI: 58.81–347.37). Those collected on SICU/ICU wards showed 9.98-fold higher risk (95% CI: 5.05–19.72) than general wards. Regarding intervention efficacy, pre-TAT overtime rates decreased by 58.6% (13.48% → 7.55%, *P* < 0.01). Contamination rate decreased by 59.8% (5.67% → 2.28%, *P* < 0.01). The median pre-TAT decreased by 15.9% (44 → 37 min, *P* < 0.01).

**Discussion:**

The joint model effectively identified workflow bottlenecks. Targeted interventions (dynamic transport scheduling, standardized training, and IoT alert systems) significantly optimized pre-TAT and specimen quality, providing a framework for improving clinical laboratory processes.

## 1 Introduction

In the field of medical laboratory testing, the timeliness of obtaining test results exerts direct and critical impacts on clinical decision-making and patient prognosis (1–3). As a core diagnostic method for urinary tract infections (UTIs), prolonged pre-analytical turnaround times (pre-TATs) for urine culture have become a pressing operational challenge. When the pre-TAT exceeds 2 h, pathogen viability significantly decreases at room temperature. For example, a 37% reduction in the survival rate of Neisseria gonorrhoeae has been observed under these conditions (*P* < 0.01) (4–6). Under these conditions false-negative rates increase by 12.3%, which may cause misdiagnosis and antibiotic misuse (7–9). Moreover, contamination rates rise from 38.6% to 50.9% (*P* < 0.01) (10).

Although numerous optimization strategies exist for reducing overall urine culture TAT, significant limitations persist. First, existing research has failed to effectively decouple delay factors across workflow stages (collection/transport/reception), resulting in a lack of quantitative analyses of each stage's impact. Second, studies have insufficiently addressed the effects of context-specific factors, such as ward operational patterns (e.g., ICU nursing peaks) and temporal characteristics (e.g., night-shift transitions) (11). Additionally, traditional regression models face methodological limitations when processing high-dimensional categorical variables (e.g., 37 ward units) (12), which hinders precise analysis of numerous contributing factors.

To address these issues, in this study, we established dual-stage analytical framework: Stage 1: K-means clustering is used to analyze wards and time periods to identify groups with similar delay patterns, such as (1) “SICU/ICU high-risk cluster” and (2) “04:00–05:59/10:00–11:59 peak interval”; Stage 2: Binary logistic regression is used to quantify odds ratios (ORs) for key factors to pinpoint dominant bottlenecks prolonging pre-TAT. Although this logistic regression-clustering joint model was successfully applied in emergency laboratory optimization (13), its effectiveness in multi-stage microbiology pre-TAT analysis remains unverified.

Accordingly, this study aimed to achieve two primary objectives: (1) to develop and validate the logistic regression-clustering joint model for quantitative analysis of multi-stage workflow bottlenecks in urine culture pre-TAT prolongation (>115 min); (2) to evaluate the practical effectiveness of model-derived interventions—including dynamic transport scheduling, standardized training, and IoT alert systems—in reducing TAT and contamination rates.

## 2 Materials and methods

### 2.1 Background information

This study was conducted at a tertiary Grade A general hospital in China with 1,700 approved beds. The clinical laboratory obtained ISO 15189 accreditation for medical laboratory quality and competence in 2014, with all testing processes strictly adhering to internationally standardized operating protocols.

**Figure 1:**
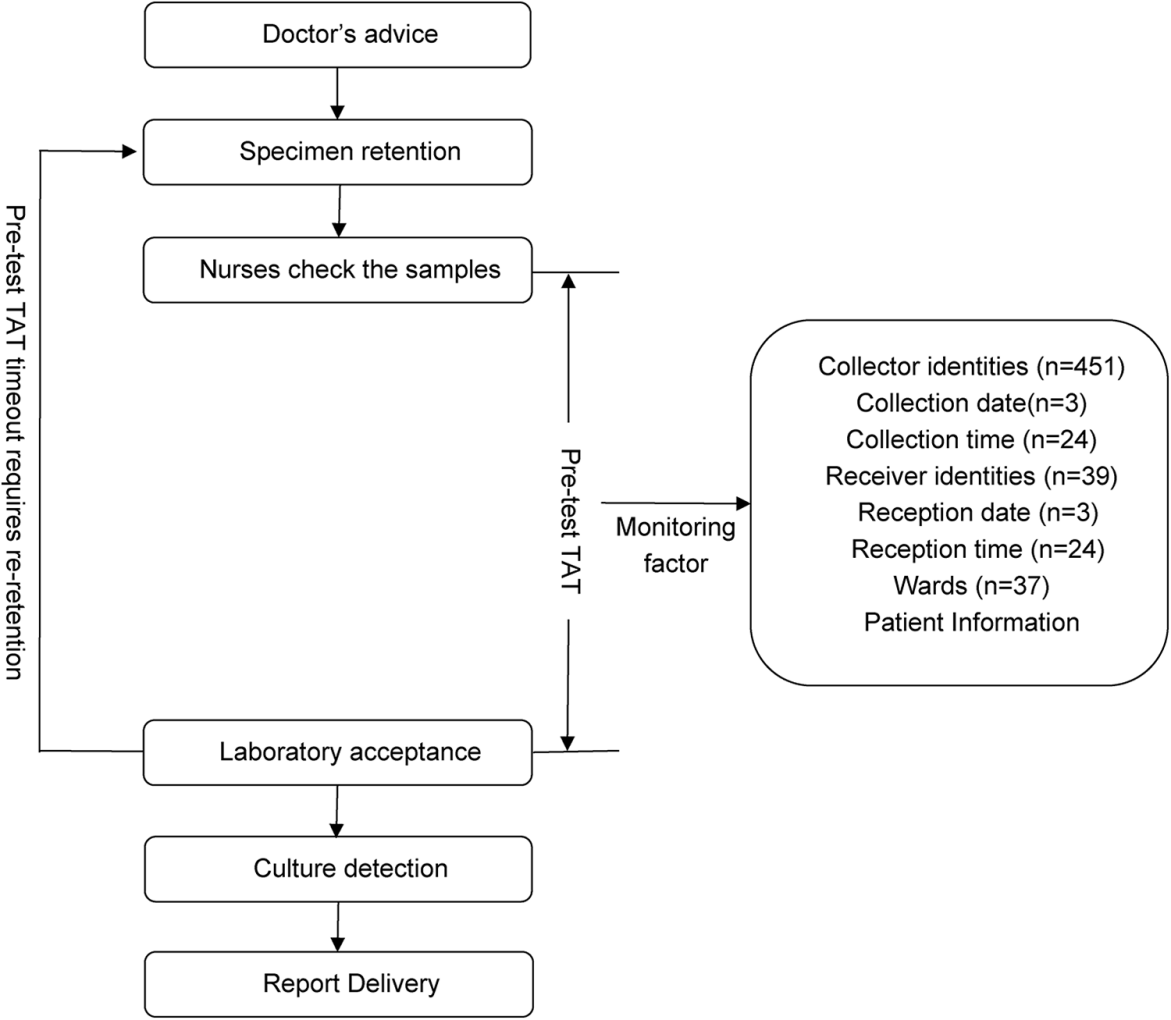
Flow chart of urine culture samples obtained from the ward.

According to *Specimen Collection and Transportation Guidelines for Clinical Microbiological Testing* and recommendations from the Clinical and Laboratory Standards Institute (CLSI) guidelines GP41-A7 (14), urine specimens should be delivered to microbiology laboratories within 2 h of collection to avoid false-negative/positive results caused by bacterial overgrowth *in vitro*, death of fastidious organisms (e.g., *Neisseria gonorrhoeae*), or degradation of formed elements (leukocytes and casts) (14, 15). Based on the hospital's logistical constraints (including transport distance, frequency, and cold chain coverage), this study defined the pre-analytic turnaround time (pre-TAT) standard for urine culture as ≤115 min. This threshold was established using dual evidence-based rationale as follows: 1) Compliance with the CLSI GP41-A7 guidelines, which mandate a 2-h transport limit (14); 2) Institutional workflow validation: Empirical data from hospital logistics audits confirmed a median transport time of 5 min (IQR: 2–8 min) for urine specimens (2023).Thus, the integration of clinical guideline adherence and institution-specific workflow parameters resulted in ≤115 min being established as a scientifically validated pre-TAT threshold. Retrospective analysis revealed that during the first quarter of 2024, the median pre-analytical TAT for urine cultures across all wards was 44 min (IQR 23–75), with an overtime rate (TAT > 115 min) of 13.48% (*n* = 1,343). Through process mapping, this study systematically outlined the end-to-end workflow from specimen collection to laboratory reception across wards (Figure 1), identifying quantifiable risk factors, including patient demographics, ward location, collection date/time/personnel, and reception date/ time/ personnel.

In the non-interventional retrospective analysis, all data were anonymously extracted from the Hospital Information System (HIS) and Laboratory Information System (LIS), excluding patient identifiers. The study protocol was approved by the Institutional Review Board of Dongyang People's Hospital (approval no. 2025-YX-048). Given the absence of patient intervention and complete data anonymity, the IRB waived the requirement for informed consent. The study complied with the principles of the *Declaration of Helsinki*.

### 2.2 Research methods

#### 2.2.1 Data collection and processing

This study adopted a retrospective cohort design; pre-intervention data from urine culture specimens were collected on wards between January and March 2024 (*n* = 1,343) and post-intervention data from specimens were collected between May and July 2024 (*n* = 1,456). Table 1 shows the basic data of the specimens. Data were extracted from the LIS and structured nursing Electronic Medical Records (EMR).

**Table 1:** Basic information of urine culture samples obtained on the ward.

Factor	Pre-intervention (*n* = 1,343)	Post-intervention (*n* = 1,456)	t/*χ*²/Z	*P*
Sex (F/M), *n*	730/613	840/616	3.16	0.08
Age, year	64.60 ± 21.34	64.83 ± 19.63	7.39	< 0.01**
Pre-TAT, min	44 (23, 75)	37 (21, 66)	−4.31	< 0.01**
Classification ward pre-TAT, min				
Ward cluster 1	115.5 (60–180)	53 (29–99)	−5.06	< 0.01**
Ward cluster 2	44 (23–75)	37 (21–66)	−4.06	< 0.01**
Ward cluster 3	37 (21–62)	33 (19–61)	−2.16	0.03*
Pre-TAT at different sampling times, min				
Collection time 1	169 (146–187.50)	152 (129–197)	−1.56	0.12
Collection time 2	41 (22–68)	36 (20–64)	−3.44	< 0.01**

Pre-TAT, pre-analytical turnaround time; Ward cluster 1, Surgical Intensive Care Unit and Intensive Care Unit; Ward cluster 2, Rehabilitation, Hematology, Oncology Surgery, Hepatobiliary Surgery, Orthopedics, Gynecology, Emergency Observation Area, Endocrinology, Brain Surgery, Vascular Surgery, Neurology, Respiratory, Obstetrics, Ophthalmology; Ward cluster 3; remaining wards; Collection time1, Specimen sampling time within 4:00–4:59 or 5:00–5:59 or 10:00–11:59; Collection time 2, other sampling time.

** *P* < 0.01.

* <0.05.

The inclusion criteria were: 1. Inpatient clean-catch midstream urine, catheterized urine, or suprapubic bladder aspiration specimens, collected as per *Technical Specifications for Specimen Collection and Transportation in Clinical Microbiology Testing* (WS/T 640—2018), including aseptic techniques and container integrity, with laboratory quality control approval; 2. Complete documentation.

The exclusion criteria were: (1) Duplicate submissions: Only the first specimen was retained if multiple specimens from the same patient were submitted within 24 h; (2) Non-standard collection: (1) Clean-catch midstream urine: Failure to clean the urethral meatal or discard initial urine; (2) Catheterized specimens: Non-sterile catheter kits or procedural contamination (based on nursing records); (3) Suprapubic specimens: Turbid aspirates or suspected skin flora contamination (per procedure notes and laboratory feedback); (3) Process deviations: Non-standard submissions due to emergencies, equipment failures, or force majeure (per nursing handover records and laboratory incident logs).

Based on the CLSI guidelines GP41-A7 and hospital monitoring factors, the following variables, which may affect pre-analytical TAT, were extracted: patient age, sex, collector nurses, collection time (hourly intervals with 24 categories), collection date, laboratory receiver person, reception time, and reception date.

pre-TAT calculation: calculated in minutes (collection time to laboratory reception time).

The pre-intervention/post-intervention timeout rate was calculated as follows: timeout rate = (number of specimens at pre-TAT > 115 min/total number of valid samples) × 100.

The pre-/post-intervention contamination rates were calculated as follows: contamination rate = (contaminated specimens/total valid specimens) × 100%.

The contamination criteria followed the CLSI guideline M41-A and WS/T 640—2018 revisions (15, 16): Clean-catch midstream urine: ≥3 microbial species, each with colony counts <10^4 CFU/ml; Catheterized/suprapubic specimens: ≥2 microbial species (excluding confirmed multidrug-resistant infections).

### 2.3 Statistical analysis

Statistical analyses were performed using the R software (version 4.1.2). Normally distributed continuous variables are expressed as the mean ± standard deviation (*x¯* ± *s*) and were compared using the *t*-test. Non-normally distributed continuous variables are presented as medians (Q1, Q3) and were analyzed using the Wilcoxon rank-sum test. Categorical and ordinal variables were summarized as counts (%), with categorical variables were compared using the chi-square test, and ordinal variables were analyzed using the Wilcoxon rank-sum test. For collinearity analysis, variance inflation factor (VIF) >10 indicated significant collinearity. After univariate analysis and collinearity screening, the selected variables were incorporated into a logistic regression model. To address potential small-sample bias or separation issues, we implemented Firth's penalized likelihood regression to optimize logistic regression outcomes. This approach reduces extreme OR estimation bias and enhances parameter stability.

For high-dimensional categorical variables (37 ward units, 24 time windows), K-means clustering was employed with silhouette scoring to determine optimal cluster numbers. Scores approaching 1 indicate superior clustering (near −1 indicates poor separation), effectively reducing model complexity and improving clinical actionability of the regression results.

## 3 Pre-intervention data analysis

### 3.1 Multicollinearity assessment

**Figure 2:**
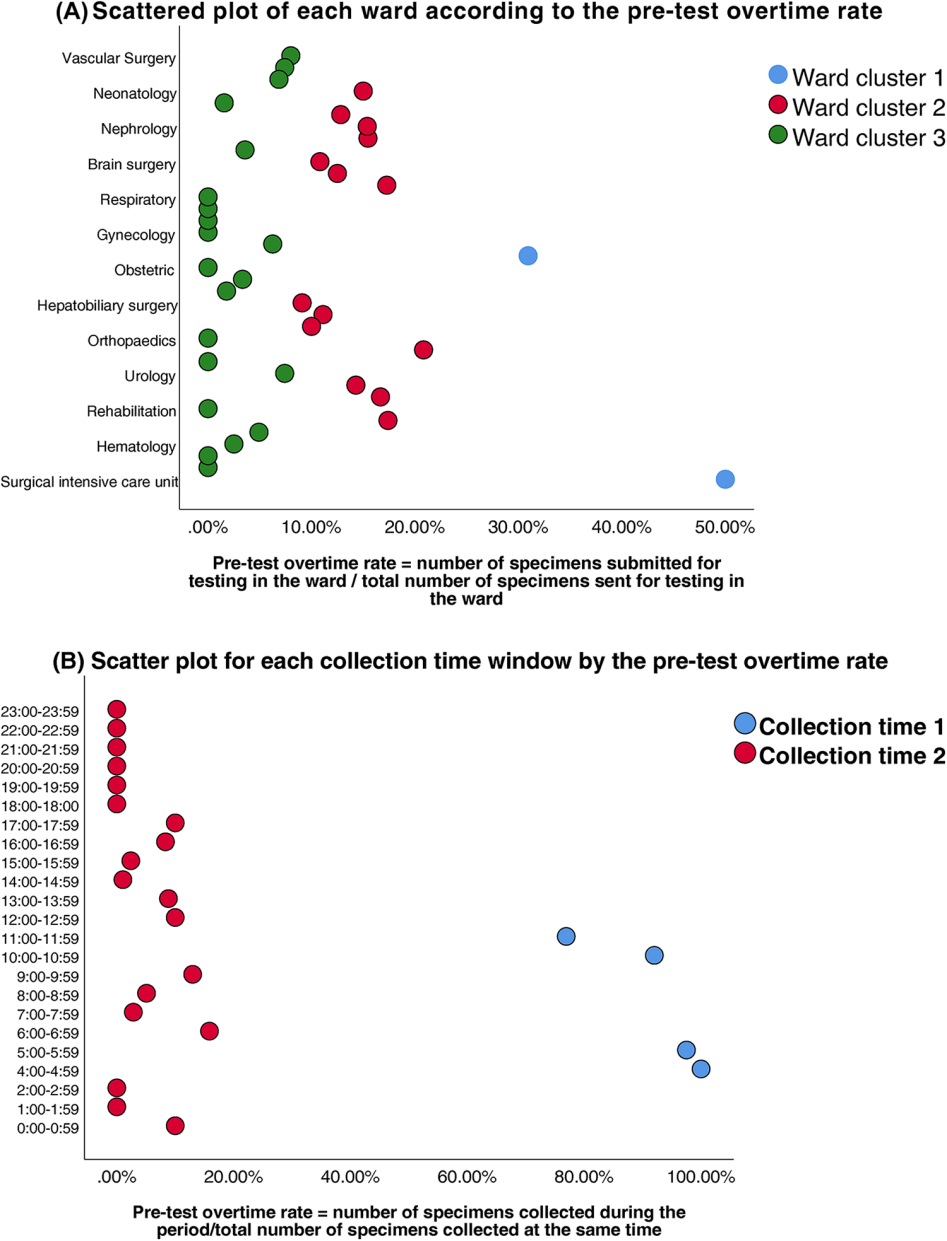
**(A)** Scatter plot of each ward according to the pre-test overtime rate. **(B)** Scatter plot for each collection time window by the pre-test overtime rate.

Prior to modeling, VIF analysis was performed to assess multicollinearity among the predictors, with group allocation as the dependent variable. Reception date and reception time window exhibited severe collinearity (VIF >10) (Table 2). Thus, reception-related temporal variables (date and time windows) were excluded. While the collection time window demonstrated moderate collinearity (VIF = 9.75), it retained statistical significance in subsequent logistic regression (OR = 142.92, 95%CI:58.81–347.37, *P* < 0.01) and represented a clinically actionable monitoring factor. Thus, collection time window data was retained for subsequent analyses. K-means clustering was applied to further classify the 37 clinical units and 24 collection time windows based on pre-analytical overtime rates. Cluster quality was validated through silhouette scoring (ward clusters: 0.72; time window clusters: 0.68), which demonstrated strong inter-cluster separation (>0.5 threshold) (Table 3). The final groupings comprised: Ward Cluster 1 (High Delay Risk): Surgical Intensive Care Unit (SICU) and Intensive Care Unit (ICU); Ward Cluster 2 (Medium Risk): 14 departments including Rehabilitation and Hematology (silhouette score: 0.61); Ward Cluster 3 (Low Risk): Remaining wards (Figure 2A); Time Window Group 1 (Peak Period): 04:00–05:59 and 10:00–11:59; Time Window Group 2 (Routine Period); and all other time intervals (Figure 2B). The final binary logistic regression model incorporated 10 clinically and statistically significant predictors: ward cluster (1–3), collector identity, collection date, collection time window (sampling times 1–2), receiver identity, and patient demographics (sex and age). Table 4 presents the variable coding schemes and reference categories.

**Table 2:** Collinear analysis of independent variables of the urine culture specimens obtained from the wards.

Variable	Collinearity statistics		t	*P*
	Tolerance	VIF		
Age	0.97	1.03	0.36	0.73
Sex	0.98	1.02	0.93	0.35
Ward	0.97	1.03	−2.63	0.00**
Collection time window	0.10	9.75	−24.41	0.00**
Reception time window	0.10	>10	23.68	0.00**
Reception date	7.46	>10	−20.65	0.00**
Collection date	0.99	1.01	0.85	0.39
Collector identity	0.98	1.03	2.65	0.00**
Receiver identity	0.98	1.02	0.18	0.86

Dependent variable: Turnaround time before test; VIF, variance inflation factor.

** *P* < 0.01.

* <0.05.

**Table 3:** Validation of optimal cluster configurations using silhouette analysis.

Cluster type	Entities	Optimal k	Rationale for silhouette score	Calculated value
Ward units	37 units	3	High separation between SICU/ICU vs. general wards ( Figure 2A )	0.72 (strong)
Collection time	24 windows	2	Clear dichotomy: peak (04–06/10–12) vs. non-peak ( Figure 2B )	0.68 (strong)
SICU nurses	36 personnel	2	Bimodal distribution (Nurse A vs. a, Figure 3A)	0.65 (medium)
SICU time windows	15 periods	2	Peak (04–08/10–12) vs. non-peak ( Figure 3B )	0.71 (strong)
ICU nurses	35 personnel	2	Clear outlier group (Nurse C vs. c, Figure 4A)	0.63 (medium)
ICU time windows	13 periods	2	Peak (04–06/10–12) vs. non-peak ( Figure 4B )	0.69 (strong)

Score interpretation: >0.60: Strong cluster structure; 0.50–0.65: Medium structure; <0.50: Weak separation (not observed).

**Table 4:** Assignment and variables of the overtime factor of turnaround time before the examination of urine culture specimens on the wards.

Name	Meaning	Assignment
Y-TAT	Endpoint variable, time of specimen sampling from nurse to laboratory receipt	Pre-TAT ≤ 115 min = 0; Pre-TAT > 115min = 1
X-sex	–	Female = 0; Male = 1
X-age	–	Continuous variable in years
X-collector identity	Specimen collection nurse	categorical variable
X-collection date	Month of specimen collection	Categorical variables, January = 0, February = 1, March = 2
X-collection time	Specimen collection time, hourly segments	Specimen sampling time within 4:00–5:59 or 10:00–11:59 = 0; rest of the period = 1
X-ward	Patient's ward	Ward cluster 1 = 0; Ward cluster 2 = 1; Ward cluster 3 = 3
X-receiver identity	Laboratory specimen receivers	Categorical variable

Pre-TAT, pre-analytical turnaround time; Ward cluster 1, Surgical Intensive Care Unit and Intensive Care Unit; Ward cluster 2, Rehabilitation, Hematology, Oncology Surgery, Hepatobiliary Surgery, Orthopedics, Gynecology, Emergency Observation Area, Endocrinology, Brain Surgery, Vascular Surgery, Neurology, Respiratory, Obstetrics, Ophthalmology; Ward cluster 3; remaining wards.

### 3.2 Logistic regression analysis of factors associated with prolonged pre-analytical turnaround time

Multivariate logistic regression analysis identified the collection time window as the most significant predictor of prolonged pre-TAT (pre-TATs >115 min) for urine culture testing in hospital wards. Specimens collected in the early morning (04:00–05:59) and peak daytime hours (10:00–11:59) demonstrated the strongest association with pre-TAT overages (*β* = 4.96, *P* < 0.01; OR = 142.92, 95%CI:58.81–347.37), followed by specimens from the SICU and ICU (*β* = 2.30, *P* < 0.01; OR = 9.98, 95%CI:5.05–19.72) (Table 5). Despite the modest sample size in critical time windows (*n* = 71), Firth's penalized likelihood regression confirmed robust effect estimates, yielding similarly extreme OR magnitudes (OR = 132.41, 95% CI:54.83–334.12). This finding aligns with clinically observed workflow disruptions, demonstrating strong model reliability for high-risk period identification.

**Table 5:** Logistic analysis of factors influencing the overtime of turnaround time before testing of the urine culture specimens on the ward.

Factor	Time out group (*n* = 181)		Not time out group (*n* = 1,162)	Univariate analysis			Multivariate analysis		
	Pre-TAT > 115 min		Pre-TAT ≤ 115 min	B	*P*	OR (CI)	B	*P*	OR (CI)
Sex (F/M), *n*		82/99	648/514	−0.42	0.00**	0.66 (0.48–0.90)	−0.27	0.32	0.77 (0.45–1.30)
Age, year		64.07 ± 1.56	64.35 ± 0.71	0.00	0.63	0.99 (0.99–1.01)	−0.01	0.31	0.99 (0.98–1.01)
Number of collector identities, *n*		111	428	0.00	0.00**	1.00 (1.00–1.00)	0.00	0.45	1.00 (1.00–1.00)
Number of Receiver identities, *n*		46	38	0.00	0.73	1.00 (1.00–1.00)	0.00	0.17	1.00 (1.00–1.00)
Number of specimens collected during this period, *n*		–	–	–	0.96	–	–	0.23	–
January		67	470	−0.05	0.80	0.95（0.65–1.39）	−0.35	0.27	0.70 (0.38–1.32)
February		59	325	0.19	0.34	1.21（0.82–1.80）	0.21	0.52	1.23 (0.66–2.31)
Number of specimens collected during this period, *n*		–	–	–	0.00**	–	–	0.00**	–
Collection time 1		68	3	5.45	0.00**	232.48 (71.99–750.79)	4.96	0.00**	142.92 (58.81–347.37)^a^
Number of specimens collected in this ward, *n*		–	–	–	0.00**	–	–	0.00**	–
Ward cluster 1		49	49	2.44	0.00**	11.43 (7.28–11.95)	2.30	0.00**	9.98 (5.05–19.72)
Ward cluster 2		43	96	1.63	0.00**	5.12 (3.36–7.79)	1.21	0.06	3.34 (1.68–6.63)

Pre-TAT, pre-analytical turnaround time; Collection time 1, specimen sampling time within 4:00–5:59 or 10:00–11:59; Ward cluster 1, Surgical Intensive Care Unit and Intensive Care Unit; Ward cluster 2, Rehabilitation, Hematology, Oncology Surgery, Hepatobiliary Surgery, Orthopedics, Gynecology, Emergency Observation Area, Endocrinology, Brain Surgery, Vascular Surgery, Neurology, Respiratory, Obstetrics, Ophthalmology; B, regression coefficient; OR, odds ratio; CI, confidence interval.

^a^ Firth's penalized likelihood regression confirmed the extreme OR magnitude (OR = 132.41, 95% CI:54.83–334.12, *P* < 0.001).

** *P* < 0.01.

* < 0.05.

To identify the ward-specific contributors to pre-TAT delays, we conducted a stratified analysis of urine culture workflows in the SICU and ICU. The key variables included the collector identity and collection time window.

### 3.3 Logistic regression analysis of pre-TAT overtime factors in the SICU and ICU

**Figure 3:**
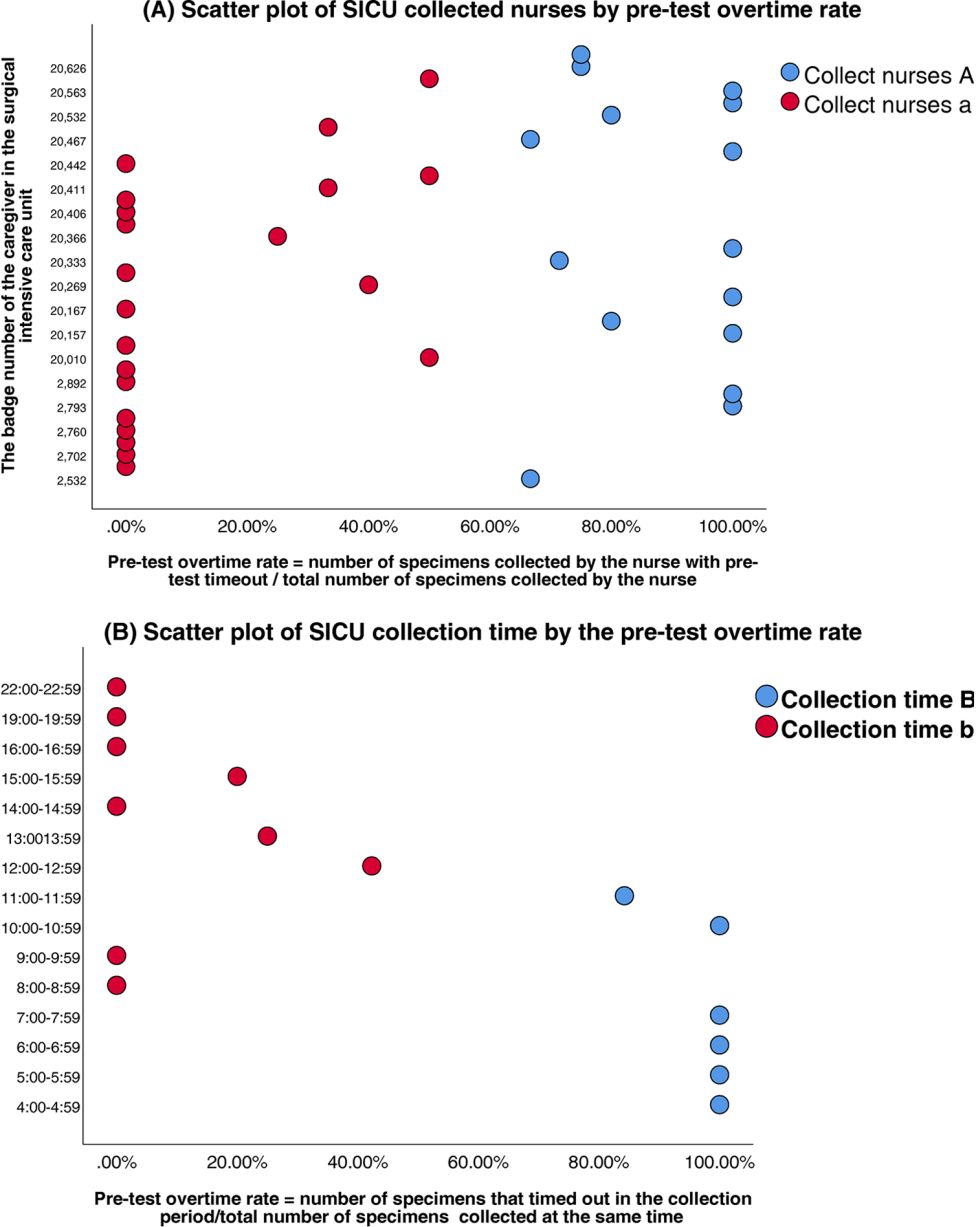
**(A)** Scatter plot of SICU collected nurses by pre-test overtime rate. **(B)** Scatter plot of SICU collection time by the pre-test overtime rate.

**Figure 4:**
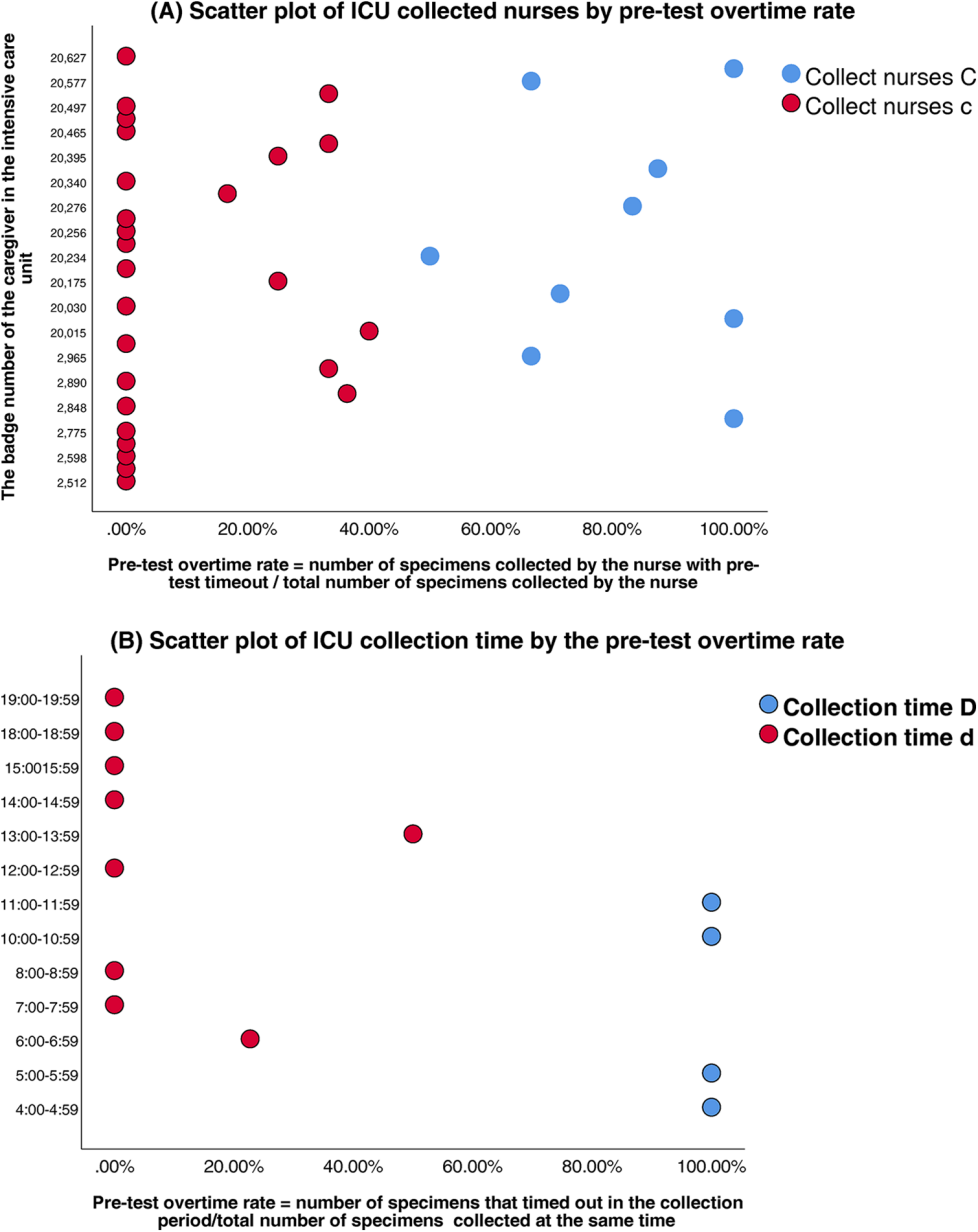
**(A)** Scatter plot of ICU collected nurses by pre-test overtime rate. **(B)** Scatter plot of ICU collection time by the pre-test overtime rate.

Clustering analysis was performed to classify healthcare personnel and collection time windows in the SICU and ICU. Silhouette scores confirmed robust cluster structures (SICU personnel: 0.65; SICU time windows: 0.71; ICU personnel: 0.63; ICU time windows: 0.69) (Table 3). The classifications were SICU Classifications: (1) Personnel clusters: Nurse Group A (*n* = 15, silhouette = 0.62); Nurse Group a (*n* = 21, silhouette = 0.67) (Figure 3A). (2) Time window clusters: Collection Period B (Peak: 04:00–07:59 & 10:00–11:59); and Collection Period b (Non-peak) (Figure 3B). ICU Classifications: (1) Personnel clusters: Nurse Group C (*n* = 6, silhouette = 0.59); Nurse Group c (*n* = 29, silhouette = 0.65) (Figure 4A). (2) Time window clusters: Collection Period D (Peak: 04:00–05:59 & 10:00–11:59); and Collection Period d (Non-peak) (Figure 4B).

Multivariate logistic regression revealed that the collection time window significantly predicted pre-analytical TAT prolongation in both units.

SICU: peak-period collections (04:00–07:59 and 10:00–11:59) showed the strongest association with delays (*β* = 2.67, *P* < 0.01; OR = 14.38, 95%CI:3.54–58.40); nurse A personnel as the collectors was a secondary predictor (*β* = 1.79, *P* < 0.01; OR = 5.96, 95%CI:2.78–19.94).

ICU: peak-period collections (04:00–05:59 & 10:00–11:59) carried the highest risk (*β* = 4.86, *P* < 0.01; OR = 128.59, 95%CI:23.43–705.63). Given the small sample size in the non-overtime group (*n* = 6), Firth's penalized likelihood regression was performed as a sensitivity analysis, the adjusted OR was 85.40 (95% CI: 18.25–399.55), confirming its robust association with pre-TAT delays (*P* < 0.001); nurse C personnel emerged as secondary predictors (*β* = 3.19, *P* < 0.01; OR = 24.33, 95%CI:5.18–114.13).

Complete regression coefficients and statistical details are provided in Tables 6A,B.

**Table 6A:** Logistic analysis of factors for the overtime of turnaround time before testing the urine culture specimens in SICU.

Factors of SICU	Time out group (*n* = 67)	Not time out group (*n* = 9)	Multivariate analysis		
	Pre-TAT > 115 min	Pre-TAT ≤ 115 min	B	*P*	OR (CI)
Number of collection nurses A, *n*	34	6	1.79	0.00**	5.96 (2.78–19.94)
Number of collections B, *n*	33	3	2.67	0.00**	14.38 (3.54–58.40)

Pre-TAT, pre-analytical turnaround time; Collection nurses A, 15 specimen sampling personnel in the ICU; Collection time B, Specimen sampling times within 4:00–7:59 or 10:00–11:59 or 13:00–13:59.

** *P* < 0.01.

**Table 6B:** Logistic analysis of factors influencing the overtime of turnaround time before the testing of the urine culture specimens in ICU.

Factors of ICU	Time out group (*n* = 58)	Not time out group (*n* = 6)	Multivariate analysis		
	Pre-TAT > 115 min	Pre-TAT ≤ 115 min	B	*P*	OR (CI)
Number of collection nurses C, *n*	25	4	3.19	0.00**	24.33 (5.18–114.13)
Number of collection time D, *n*	33	2	4.86	0.00**	128.59 (23.43–71.63)^a^

Pre-TAT, pre-analytical turnaround time; Collect nurses C, 6 specimen sampling personnel in the ICU; Collection time D, Specimen sampling time within 4:00–5:59 or 10:00–11:59.

^a^ Firth's penalized likelihood regression confirmed the extreme OR magnitude (OR = 85.40, 95%CI: 18.25–399.55, *P* < 0.001).

** *P* < 0.01.

## 4 Improvement measures

### 4.1 Enhancement of specimen collection personnel

The following strategies may improve specimen collection:

1) Stratified Training Program: Develop a tiered training plan (theory + practice) based on the *Specimen Collection and Transport Standards for Clinical Microbiological Testing* (WS/T 640—2018) (15) and CLSI guideline GP41-A7[National Health Commission of the People's Republic of China (NHC), n.d.]. The key focus areas include using aseptic techniques for clean-catch midstream urine collection (e.g., urethral orifice disinfection methods and the proportion of initial urine to be discarded); sterile catheterization procedures (e.g., WHO hand hygiene protocols and catheter insertion depth control); and developing patient education strategies (e.g., communication approaches for elderly or cognitively impaired patients).
2) Monthly Quality Audits: Random selection of 10% of the urine specimens for quality assessment (e.g., contamination rate and label completeness), with results incorporated into the nursing department performance evaluations.
3) Closed-Loop Management System: Establish a “specimen quality feedback-correction-recheck” workflow within the LIS.
4) Standardized Urine Collection Kits: Providing pre-assembled kits containing sterile gloves, disinfectant wipes, disposable sterile urine cups, and transport tubes with boric acid preservatives to minimize preparation time.

### 4.2 Enhancement of specimen collection timing

The following approaches may improve specimen collection timing:

1) Peak-Hour Staffing Optimization: Identify peak specimen submission periods in major wards and dynamically allocate dedicated transport personnel (≥1 per ward per shift) via scheduling software.
2) Aseptic Transport Upgrades: Replace traditional open urine cups with closed sterile transport devices (e.g., BD Urine Monovette®). During low-demand periods (e.g., midday/night shifts), pneumatic tube system (PTS) transport should be prioritized.
3) Pre-TAT Alert Module: Integrate a “specimen timeout warning system” into the LIS to automatically notify the relevant nurses when the pre-TAT exceeds 90 min.
4) Daily Pre-TAT Compliance Reports: Generate ward-specific pre-TAT compliance rate reports and discuss results during morning shift handovers.
5) Refrigerated Temporary Storage: Install 2–8°C refrigerators in ward specimen holding stations for samples when delayed transport is unavoidable.

## 5 Pre-analytical TAT and contamination rate outcomes

Following the implementation of quality improvement interventions, significant reductions in the pre-analytical workflow metrics were observed. The median pre-TAT time for urine culture processing significantly decreased from 44 min (IQR: 23–75 min) to 37 min (IQR: 21–66 min) (Z = 4.31, *P* < 0.01). The overall pre-analytical timeout rate decreased from 13.48% to 7.55% (*χ*² = 26.30, *P* < 0.01), determined using the Wilcoxon rank-sum test. Stratified analyses revealed pronounced improvements in SICU and ICU sample rates.

In the SICU, the timeout rate decreased by 58.88% (50.00% vs. 20.56%; *χ*² = 19.58, *P* < 0.01), and the pre-TAT of urine cultures collected by nursing staff was reduced from 180 (126–223) min pre-intervention to 55 (20–130) min post-intervention (Z = −3.98, *P* < 0.001).

In the ICU, the timeout rate decreased by 74.11% (30.90% vs. 8.00%; *χ*² = 21.58, *P* < 0.01), with the pre-TAT of nursing-collected urine cultures decreasing from 152 (119–179) min to 117 (76–161) min (Z = −4.82, *P* < 0.001).

Concurrently, the overall contamination rate for urine cultures across wards decreased by 59.79%, dropping from 5.67% to 2.28% (*χ*² = 13.11, *P* < 0.01). The detailed results are presented in Table 7.

**Table 7:** Comparison of Pre-TAT and overtime of the urine culture tests in the ward before and after intervention.

Factor	Pre-intervention (*n* = 1,343)			Post-intervention (*n* = 1,456)			Z/χ²	*p*
	Pre-TAT>115 min	Pre-TAT ≤ 115 min	Timeout ratio, %	Pre-TAT>115 min	Pre-TAT ≤ 115 min	Timeout ratio, %		
Pre-TAT, min	44 (23, 75)			37 (21, 66)			−4.31	< 0.01**
Contamination rate of urine culture, %	5.67%			2.28%			13.11	< 0.01**
Number of urine culture specimens across all wards, *n*	181	1,162	13.48	110	1,346	7.55	26.30	< 0.01**
Number of urine culture specimens collected at collection time 1, *n*	68	3	95.77	37	8	82.22	5.89	0.02*
Number of urine culture specimens in the SICU ward, *n*	49	49	50	22	85	20.56	19.58	< 0.01**
Number of specimens collected by SICU time period B, *n*	33	3	91.67	18	7	72	4.16	0.04*
Number of specimens collected by SICU nurses A, *n*	34	6	85	5	12	29.4	17.06	< 0.01**
Pre-TAT for SICU collection A, min	180 (126, 223)			55 (20, 130)			−3.98	< 0.01**
Number of urine culture specimens in the ICU ward, *n*	43	96	30.9	10	115	8	21.58	< 0.01**
Number of specimens collected in the ICU time period D, *n*	33	2	94.3	6	7	46.2	14.42	< 0.01**
Number of specimens collected by ICU nurses C, *n*	25	4	86.2	1	21	4.5	33.38	< 0.01**
Pre-TAT for ICU collection C, min	152 (119, 179)			117 (76, 161)			−4.82	< 0.01**

Pre-TAT, pre-analytical turnaround time; Collection time 1, Specimen sampling times within 4:00–5:59 or 10:00–11:59; Collection time B, Specimen sampling times within 4:00–7:59 or 10:00–11:59; Collection time D, Specimen sampling times within 4:00–5:59 or 10:00–11:59 or 13:00–13:59; Collect nurses A, Fifteen sampling nurses with high urine culture overtime rates in the SICU; Collect nurses C, Six sampling nurses with high urine culture overtime rates in the ICU; Timeout rate = (number of specimens at pre-TAT > 115 min/total number of valid samples) × 100%; Contamination rate = (number of contaminated specimens/total number of valid specimens sent for testing) *100%, and the criteria for determining contaminated specimens: Bacteria ≥3 in clean mid-stream urine specimens with colony counts <10^4 ^CFU/ml for each microorganism; Bacteria ≥2 in catheterization or cystocentesis specimens (except for definitive multidrug-resistant bacterial infections).

** *P* < 0.01.

* <0.05.

## 6 Discussion

### 6.1 Key findings and clinical implications

As a cornerstone diagnostic technique for UTIs, urine culture testing efficacy is significantly compromised by prolonged pre-TATs. Our logistic regression-clustering joint model systematically quantified two dominant bottlenecks: temporal patterns (specimens collected during 04:00–05:59 and 10:00–11:59 exhibited extreme delay risk; OR = 142.92, 95% CI: 58.81–347.37), which is primarily attributable to nursing workload conflicts (e.g., shift transitions, medication rounds) (17); and ward-specific workflows (SICU/ICU samples showed 9.98-fold higher delay odds compared with those of general wards; 95% CI: 5.05–19.72), due to competing clinical priorities (e.g., ventilator adjustments) and inexperience in specimen handling.

### 6.2 Intervention efficacy and workflow optimization

Model-derived targeted interventions achieved significant improvements: dynamic resource allocation, in which peak-hour staffing optimization reduced pre-TAT overtimes by 14.15 percentage points (95.77%→82.22%, *P* = 0.02). Standardized training resulted in a decrease in the median pre-TAT by 69.4% (180→55 min, *P* < 0.01) in SICUs and 23.0% (152→117 min, *P* < 0.01) in ICUs. Moreover, in IoT-enabled systems, smart alerts and refrigerated storage reduced contamination rates by 59.8% (5.67%→2.28%, *P* < 0.01). These align with lean management principles validated in Korean laboratories (18, 19), enhancing operational efficiency and diagnostic reliability.

### 6.3 Generalizability and uncontrolled confounders

While interventions reduced pre-TAT overtimes by 43.9%, three contextual factors require consideration: workflow heterogeneity, unmeasured confounders, EMR downtimes, and resource disparities. Regarding workflow heterogeneity, manual transport systems may respond differently to IoT alert systems than automated pneumatic networks. Unmeasured confounders, nursing task saturation during peak hours potentially delays specimen labeling. Moreover, EMR downtimes (0.3% of collections) artificially prolong recorded TAT (20, 21). Finally, due to resource disparities, hospitals lacking cold-chain infrastructure may achieve smaller contamination reductions.

### 6.4 Limitations and future research directions

The limitations of this study include its single-center design (pre-/post-intervention *n* = 1,343/1,456), which limits generalizability to resource-constrained settings; the extreme OR for peak periods (OR = 142.92, *n* = 71) requires cautious interpretation despite robustness checks via Firth's bias-correction; and its retrospective nature, which prevented controlling for environmental variables (e.g., transport temperature/humidity). Additionally, we only focused on temporal/personnel factors, omitting systemic issues, like EMR interoperability.

### 6.5 Future research prospectives

Future studies should employ multicenter prospective designs with IoT environmental sensors; develop dynamic risk-prediction models incorporating real-time workload metrics; and validate interventions across various hospital tiers (primary care vs. tertiary hospitals).

## 7 Conclusion

The logistic regression-clustering joint model effectively identified critical pre-analytical bottlenecks, which can inform the development of targeted interventions. Implementation of dynamic shift scheduling (peak hours: 04:00–05:59 & 10:00–11:59), Intelligent early warning system systems, and standardized protocols significantly reduced pre-TAT overtimes by 43.9% and contamination rates by 59.8%. Hospitals should integrate these evidence-based strategies with real-time TAT dashboards for sustainable quality improvement.

## Data Availability

The original contributions presented in the study are included in the article/Supplementary Material, further inquiries can be directed to the corresponding author.
